# Phenomenological Study on the Lived Experience of Patients After Major Cardiac Surgery in Intensive Care Units

**DOI:** 10.1111/nicc.70134

**Published:** 2025-08-08

**Authors:** Caterina Mercuri, Vincenzo Bosco, Assunta Guillari, Teresa Rea, Patrizia Doldo, Rita Nocerino, Vincenza Giordano, Silvio Simeone

**Affiliations:** ^1^ Department of Clinical and Experimental Medicine University of Catanzaro MagnaGraecia Catanzaro Italy; ^2^ Department of Medical and Surgical Sciences University Hospital Mater Domini, Magna Graecia University Catanzaro Italy; ^3^ Department of Public Health University of Naples “Federico II” Naples Italy; ^4^ Department of Translational Medical Science University of Naples “Federico II” Naples Italy; ^5^ ImmunoNutritionLab at CEINGE – Advanced Biotechnologies University of Naples “Federico II” Naples Italy; ^6^ Department of General Surgery and Women's Health AORN Antonio Cardarelli Naples Italy

**Keywords:** cardiac surgery, intensive care unit (ICU), nursing support, patient experience, psychological impact, qualitative research

## Abstract

**Background:**

Intensive care unit (ICU) experiences after cardiac surgery significantly influence patient outcomes, including psychological disorders, quality of life and overall comfort. The existing literature emphasises psychological impacts and transitions from ICU to general wards, but there is a paucity of qualitative research focussing specifically on post‐cardiac surgery ICU experiences.

**Aim:**

This study aimed to explore the lived experiences of patients in the ICU following major cardiac surgery, providing a comprehensive understanding of their emotional and psychological challenges.

**Study Design:**

Utilising Cohen's phenomenological approach, we conducted in‐depth interviews with 20 patients from a cardiothoracic ICU in Southern Italy. Participants were chosen through purposive sampling. Data were analysed using thematic analysis to identify recurring themes.

**Results:**

Five main themes emerged from the data: (1) ‘Closing the eyes and not opening them’, highlighting pervasive fear and anxiety; (2) ‘Confusion upon awakening’, marked by disoriented memories and the fear of not being able to breathe; (3) ‘Time stood still’, describing a distorted perception of time; (4) ‘The closeness of my angels: the nurses’, underscoring the critical role of nursing support; and (5) ‘The other side: exclusion from care’, reflecting feelings of marginalisation during the care process.

**Conclusions:**

The study underscores the complex experiences of ICU patients' post‐cardiac surgery, emphasising the need for psychological support and inclusive communication strategies to enhance patient outcomes. Further research should focus on developing tailored interventions to support these patients through their recovery process.

**Relevance to Clinical Practice:**

There is a clear need for enhanced psychological support for patients before, during and after the ICU stay. Preoperative education programmes that set realistic expectations and provide coping strategies can significantly reduce anxiety and improve patient outcomes. Additionally, enhancing the role of nurses in offering emotional support and involving patients in care decisions can lead to a more positive ICU experience. Psychological interventions can optimise preoperative expectations and reduce hospital stays, offering significant cost–benefit advantages for healthcare systems.

**Impact Statements**

- What is known about this topic
  ○ Cardiac surgery patients in intensive care units (ICUs) face significant psychological and emotional challenges, including anxiety, confusion and fear of death.
  ○ Previous research has often focussed on mechanical ventilation, pain and transition to general wards, with limited in‐depth qualitative exploration of patients' lived experiences post‐cardiac surgery.
  ○ Nurses play a crucial role in providing emotional support, but patients frequently report feelings of exclusion from clinical decisions and communication.
- What this paper adds
  ○ This paper offers a phenomenological insight into the ICU experience after major cardiac surgery, highlighting five key themes including fear of dying, confusion upon awakening, altered time perception, emotional support from nurses and exclusion from care.
  ○ It reveals the critical dual role of nurses as both clinical caregivers and emotional anchors, while also identifying the need for more inclusive and patient‐centred communication.
  ○ It recommends implementing tailored preoperative education and psychological interventions to alleviate anxiety, enhance patient participation in care and improve recovery outcomes.

## 1 Introduction

The advancement of medical knowledge and the technological innovations used has led to an increase in surgical procedures globally [1, 2]. These complex surgical interventions often pose challenges that necessitate post‐operative intensive care [3, 4].

Although it is challenging to establish a global estimate of intensive care unit (ICU) admissions due to significant variability between countries, the number of ICU admissions depends largely on patient conditions, the need for advanced interventions and the availability of resources [5]. In the context of this variability, specific regional data provide insight into ICU utilisation trends. For instance, in the United States, over 16.5 million acute inpatient hospitalisations involved ICU admissions between 2006 and 2015 [6].

In South Korea, there were more than 3.5 million ICU admissions from 2010 to 2019, showing an increasing trend in both ICU admissions and the use of advanced medical interventions [7]. Similarly, ICU admissions in Hong Kong rose from 11 267 in 2008 to 14 068 in 2018, marking a 24.86% increase, compared with a population growth of just 5.71% during the same period (from 7.0 to 7.4 million) [8], indicating a notable rise in ICU utilisation relative to population growth [9].

In Europe, a survey conducted across 228 hospitals in 27 countries showed that nearly all hospitals had ICUs, but there were differences in the levels of care provided, with high‐income countries being more likely to have advanced ICUs equipped with advanced technology and resources, allowing for a higher standard of care compared to middle‐income countries. This survey recorded 5159 ICU admissions in a 2‐day period, illustrating the high ICU utilisation in Europe [10]. Furthermore, a study on intensive cardiac care units in Europe highlighted organisational differences and admission policies, reporting varying rates of ICU admissions based on the severity of the patients' conditions and the level of ICU available [10]. In addition, the experience of patients in an ICU after cardiac surgery differs significantly from that in non‐cardiac surgery ICUs, with fewer studies available to compare these environments [11].

In Italy, although ICU admissions are often influenced by chronic conditions and gender differences, these factors are less central to the current focus on post‐cardiac surgery ICU admissions. However, such information provides important context on the broader ICU landscape. An analysis of data from 2378 patients across 26 hospitals found that 16.6% required ICU care due to severe health conditions, with men being admitted more frequently than women [12].

## 2 Background/Justification for Study

Cardiovascular diseases, in particular, and severe post‐operative complications are major factors driving surgical ICU admissions. Patients requiring mechanical ventilation are particularly likely to be admitted due to the critical nature of their conditions [13, 14].

However, regardless of the ICU admission cause, the experiences of patients in intensive care after surgery influence the course of the disease, potentially leading to psychological disorders and affecting the quality of life of patients, as well as reducing specific comforts such as sleep quality, effective pain management and overall emotional well‐being [15, 16, 17]. These psychological disorders can occur in up to 55.6% of ICU patients both during and after their hospitalisation [18, 19, 20]. These disorders primarily include acute stress reactions, which can manifest as anxiety and depression, and in more severe cases, develop into post‐traumatic stress disorder (PTSD) [21, 22]. ICU patients often experience stress from the life‐threatening nature of their conditions and invasive procedures, leading to long‐term psychological issues such as anxiety and depression.

A study found that 41% of ICU survivors exhibited anxiety symptoms and 34% had PTSD symptoms 1 year after their ICU treatment [21]. Many patients recall pain, anxiety and confusion during their ICU stay, emphasising the importance of psychological and family support [23] with a significant percentage manifesting anxiety and post‐traumatic stress [24]. Negative experiences such as pain, sleep disturbances and feelings of abandonment were common. Although patients often reported feeling a sense of security from the support of nurses following transfer to general wards, they still expressed vulnerability and anxiety regarding their condition [25, 26, 27].

Although most research has focussed on experiences related to mechanical ventilation weaning [28, 29], the transition from ICU to general wards [16], pain and delirium [30] and end‐of‐life care [31], there remains limited qualitative research specifically exploring patient experiences after cardiac surgery [11, 32, 33], which is the focus of this study. Despite the fact that approximately 1.5 million cardiac surgeries are performed globally each year [34], making it a leading reason for ICU admissions [13, 35], more detailed exploration of these specific ICU experiences is needed.

The typical ICU stay after major cardiac surgery lasts up to 5 days post‐operatively [36], during which patients experience significant emotional and psychological distress [33, 37].

Studies have shown that frequent sleep interruptions, communication difficulties and other stressors during ICU stays can lead to long‐lasting distress, pain and anxiety [36, 38, 39, 40, 41]. Research on patient pain perception post‐cardiac surgery reveals that although many patients recall pain during their ICU stay, they often struggle to communicate it due to sedation and mechanical ventilation [42].

Although studies, including those conducted in Italy, have analysed aspects of the ICU experience post‐cardiac surgery, they have highlighted the need for more research into patient experiences, especially given the unique cultural and healthcare contexts [11, 39, 43, 44]. These studies stress the lack of a comprehensive understanding of the patient experience during ICU stays after cardiac surgery. This gap has led to this study, which aimed to provide a holistic view of patient experiences in intensive care after cardiac surgery.

Addressing these gaps through focussed research could significantly improve patient care and accommodate the dynamic conditions during recovery.

## 3 Aim

Therefore, the aim of this study was to investigate the experiential aspects of intensive care for patients undergoing major cardiac surgery.

## 4 Design and Methods

### 4.1 Study Setting

The study was conducted among hospitalised participants in the cardiothoracic ICU of a university hospital in Southern Italy, a regional reference centre for cardiac pathologies requiring surgical intervention. The ICU, consisting of six beds and two isolation stations, is equipped with both invasive and noninvasive monitoring systems, including ventilator support. The unit is organised as an open space, with artificial lighting present for approximately 20 h daily, and limited natural light entering through specific windows that only directly illuminate two of the stations. This setting was crucial for observing the participants' post‐operative experiences, given the specific environmental and clinical conditions present in the ICU.

### 4.2 Study Design

The methodology employed for this study follows hermeneutic phenomenology according to Cohen's approach [45].

This method combines the characteristics of Husserlian descriptive phenomenology and Gadamerian interpretative phenomenology. Although descriptive phenomenology tends to describe the general characteristics of a phenomenon, remaining free from external influencing factors, interpretative phenomenology seeks to describe, understand and interpret individual participants' experiences [46]. These experiences are inevitably influenced by social, political and cultural contexts and by surrounding individuals [47].

The phenomenological approach used allows for the analysis of phenomena from individual and subjective representations, considering the sense and meaning attributed to particular events [48, 49]. This enables an in‐depth exploration and understanding of the lived experiences of participants, focussing on issues of meaning and providing a deeper comprehension of participants' experiences and the meanings they attribute to them. This methodology is ideal for nursing research, particularly when exploring new topics [45]. This study adhered to established criteria including credibility, transferability, dependability and confirmability for reporting qualitative research standards [50].

### 4.3 Sample

A purposive sampling method was used to recruit study participants.

Participants were selected from those involved in a broader quantitative study conducted in the same ICU, based on specific inclusion criteria that ensured they had undergone major cardiac surgery and were clinically stable. The recruitment process was managed by the same research team member for both studies, facilitating trust and clear communication. Ethical considerations were carefully addressed: Participants were fully informed about the purpose and nature of both studies, and separate informed consent was obtained to respect their autonomy and avoid any undue burden or conflicts of interest. Initial contact with potential participants occurred while they were still hospitalised, with follow‐up after discharge to confirm participation and arrange interview logistics.

Inclusion criteria for participation were as follows: (a) being of legal age (≥ 18 years) according to Italian law; (b) discharged from the university hospital at least 15 days before; (c) undergoing major cardiac surgery (coronary artery bypass graft [CABG] surgery and/or valve replacement with median sternotomy); (d) ICU stay longer than 72 h; (e) clinically stable (e.g., have had a blood pressure trend within normal limits in the last 48 h of hospitalisation; have not had rhythmic alterations in the last 48 h of hospitalisation; and have recorded in the last 48 h of hospitalisation a saturation of the ambient air within normal limits without any aid from a device); (f) willingness to participate in the study; and (g) signing the informed consent. Exclusion criteria included the following: (a) refusal to participate or withdrawal from the study; (b) severe neurological impairments or significant organ failures; and (c) refusal to sign the informed consent.

### 4.4 Data Collection Tools and Methods

Data were collected between February 2024 and May 2024.

After explaining the study's nature, each participant chose the interview's time and place, ensuring they could freely recount and describe their experiences in a comfortable setting. The interviews were conducted individually within 15 days of discharge to capture early post‐operative experiences in the participants' and researchers' native language (Italian) by two trained researchers (C.M. and V.B.) using a single open‐ended question: ‘Can you describe your experience in the Cardiac Surgery Intensive Care Unit?’ Open‐ended questions allowed participants complete freedom in reporting their experiences, making their ‘world’ the investigation's focus rather than the researchers' preselected topics [51]. The participants' ‘world’ becomes the focus of the investigation rather than topics chosen by researchers [51].

Interviewers adopted an empathetic approach to encourage participants' narration, ensuring a welcoming attitude [52]. This non‐judgemental approach helped facilitate the description of their experiences. During and after the interviews, researchers also focussed on observing and documenting non‐verbal content and the chosen setting, which helped enhance the understanding of the narrative [53].

Field notes were taken during the interviews to capture reflections on the setting and language used. During and after the interview, the investigator took field notes on her own reflections, the setting, environment and patients' body language. When participants seemed not to have anything further to say, the researcher asked them whether they had anything more to add. If they did not, the interview ended. Data saturation, or redundancy of themes, was achieved after 20 interviews [45, 54]. The interviews continued until the questions did not give rise to any new relevant information to further the study purposes (i.e., data saturation). In line with Strauss and Corbin's [55] indications, it was considered that enough data saturation had been achieved based on the amount of repetition in the new data collected.

All interviews were conducted at participants' homes, in a room where only the interviewer and the participant were present. All interviews were audio‐recorded and lasted between 30 and 50 min, with an average duration of approximately 37 min. To familiarise researchers with the methodology and to help refine the interview approach, three preliminary interviews were conducted, but these were not included in the data analysis.

### 4.5 Data Analysis

Cohen's method allows a deeper knowledge of the meaning that people give to their lived experience, and rigorous analysis is enhanced by bracketing. All investigators involved in the data analysis bracketed their presuppositions before data collection. This technique of ‘critical reflection’ involves systematically documenting the researchers' assumptions and beliefs about the phenomenon under study. The purpose of this exercise is to help researchers identify and set aside personal biases, thereby minimising their influence on the data analysis process [45].

Data analysis actually began during the interviews conducted. The analysis of these data was then conducted by two researchers who conducted the interviews (C.M. and V.B.), who had no prior contact with the participants, and two other members of the research team (A.G. and S.S.). Interviews were transcribed verbatim and supplemented with contact summaries.

Each researcher first read all the interviews to get a general sense of the content, and re‐read each transcription line by line, assigning general themes to various text. This is described by Cohen as ‘immersing in the data’ process [45].

This immersion aims to create an initial interpretation guiding subsequent data coding phases. Researchers then re‐read the transcripts line by line, assigning indicative themes to various passages. The process of writing and rewriting was crucial in our data analysis. This iterative approach allowed for continuous reflection and refinement of emerging interpretations, ensuring a deeper and more accurate understanding of the participants' lived experiences. Themes and subthemes generated from participants' experiences were compared among all researchers without disagreements. Participants were informed of the identified themes in a follow‐up meeting to confirm the generated themes, with no discrepancies noted.

### 4.6 Rigour of the Study

Adherence to Lincoln and Guba's criteria further confirmed the scientific rigour of this study. Purposive convenience sampling until saturation data ensured credibility, corresponding to internal validity and ensuring the study examined its intended focus [56, 57, 58]. Dependability was ensured through triangulation, involving multiple researchers in the same analysis to provide diverse observations and conclusions [56]. This method can confirm results or offer different perspectives, adding breadth to the phenomenon of interest.

Confirmability was ensured through participant briefing and confirmation of generated themes. Providing a detailed description of participants' experiences and their socio‐demographic characteristics ensured transferability. Data analysis and result verification, as well as interview conduction, were performed in Italian. Translation and back‐translation processes followed World Health Organization (WHO) methodology for validating tools across different cultures and languages, focussing on conceptual content rather than on literal equivalence [59]. This ensured the data's meaning was preserved.

### 4.7 Ethics and Research Approvals

Ethics approval was obtained from the Ethics Committee of the Calabria Region, Central Area, with protocol number 276/2022 on 15 September 2022.

Participants were selected from a broader quantitative study conducted in the same ICU. The recruitment process was managed by the same team member to foster trust and ensure transparency. Ethical safeguards were implemented to protect participant autonomy: All individuals received complete information about the nature and scope of both studies and were asked to provide separate informed consent for participation in each. This approach minimised ethical conflicts and upheld voluntary participation in accordance with the Declaration of Helsinki. Written consent was obtained before the interviews, with explicit consent for audio recording. Collected data were kept confidential and used in compliance with current data protection and privacy regulations.

## 5 Results

Interviews were conducted with 20 patients, comprising 13 men (65%) and 7 women (35%), with an average age of 63.5 years (ranging from 42 to 77 years). Eighty percent (80%) of participants were married, with 55% having two children. Regarding education, 70% had completed middle or high school. In terms of employment status, 65% were still employed, followed by 20% who were retired. As for the type of surgery, 60% of participants underwent elective procedures, whereas 40% underwent emergency surgeries. Main socio‐demographic characteristics of the study population are reported in Table 1.

**TABLE 1:** Main socio‐demographic characteristics of study participants.

ID	Gender	Age	Number of children	Marital status	Emergency or elective intervention	Education level	Profession
AZ01	F	63	2	Married	Elective	High school	Employed
BY02	M	77	2	Married	Elective	High school	Employed
CX03	M	66	1	Married	Elective	High school	Employed
DW04	M	56	2	Married	Emergency	High school	Employed
EV05	M	49	1	Married	Elective	High school	Not employed
FU06	M	67	3	Married	Elective	Middle school	Employed
GT07	M	71	1	Married	Emergency	Middle school	Employed
HS08	F	62	2	Divorced	Emergency	Middle school	Employed
IR09	M	69	2	Divorced	Emergency	Elementary	Retired
JQ10	M	42	0	Single	Emergency	Degree	Employed
KP11	F	68	3	Widower	Elective	Middle school	Retired
LO12	M	53	—	Single	Elective	High school	—
MN13	F	55	1	Married	Emergency	High school	Not employed
NM14	F	62	2	Married	Elective	Middle school	Not employed
OL15	F	74	2	Married	Elective	Middle school	Retired
PK16	F	69	2	Married	Elective	High school	Employed
QJ17	M	64	2	Married	Emergency	Degree	Employed
RI18	M	77	2	Widower	Elective	Middle school	Retired
SH19	M	66	1	Married	Elective	Middle school	—
TG20	M	61	2	Married	Emergency	Degree	Employed

From the data analysis, the following five main themes were generated: ‘Closing the eyes and not opening them’, ‘Confusion upon awakening’, ‘Time stood still’, ‘The closeness of my angels: the nurses’ and ‘The other side: exclusion from care’.

### 5.1 THEME 1: ‘Closing the Eyes and Not Opening Them’

All participants expressed that cardiac surgery evokes fear and worry, feelings that persist from the diagnosis through the entry into the operating room and the immediate post‐operative period. These emotions were consistently reported across both elective and emergency surgeries, with no distinction in the intensity of fear experienced.

AZ 01 said:

I remember those sensations well, the fear I had, which I still partly have today. I kept thinking about the same thing: if I close my eyes as they say, will I be sure to open them again?

This persistent fear reflects a universal concern among patients facing cardiac surgery: the uncertainty of survival.

FU 06 added:

Even though everything was explained to me, they still had to operate on my heart, and that's where life is… I was always afraid that something might go wrong, afraid of not seeing my wife and children again.

These testimonies illustrate how, despite being informed, emotional distress remains dominant.

### 5.2 THEME 2: ‘Confusion Upon Awakening’ With the Sub‐Theme ‘Fear of Not Breathing’

Participants described the awakening phase as fragmented, marked by blurred and disoriented memories. Many recalled sensations rather than coherent events.

BY02 shared:

I vaguely remember waking up… it was like having a veil over my eyes… voices in the distance… a throat discomfort and then they told me to breathe, that everything had gone well… but I felt like I was seeing them from afar.

This perception underscores how the environment and sedation influenced patients' awareness.

LO 12 added:

I remember little of the moments right after the operation… everything seemed wrapped in fog… voices, air re‐entering my body not through a small hole, a tube but through the nose… a good feeling… but then I remember little else.

The recollections frequently involved fear of respiratory failure, which emerged as a distinct sub‐theme.

#### 5.2.1 Sub‐Theme: Fear of Not Breathing

Many participants described a vivid fear of being unable to breathe independently.

GT07 noted:

In that fog, I clearly remember the throat discomfort, the tube that didn't let me swallow and pressed against my throat… all accompanied by anxiety, the fear of not being able to breathe alone.

This theme not only emphasises physical discomfort but also the psychological impact of intubation.

NM 14 clarified:

…and I remember well the constant sensation of not being able to breathe… a feeling of anxiety that suddenly I would not be able to breathe on my own…

### 5.3 THEME 3: ‘Time Stood Still’

Unlike the fragmented memories of awakening, participants vividly recalled a distorted sense of time during their ICU stay. Many described a liminal state in which time ceased to exist.

DW04 stated:

Time seemed to stop, I watched the clock hands above my head, and they didn't move.

Such perceptions reflect the sensory deprivation and artificial environment typical of ICUs.

AZ 01 added:

Nights felt endless… I understood it was night only because they did certain things… I couldn't tell if it was night, morning, afternoon, day… the constant beeps of the monitors, the lights always on… you closed your eyes hoping to have slept for hours, but it was only a few minutes.

These testimonies point to the importance of environmental cues in maintaining temporal orientation.

### 5.4 THEME 4: ‘The Closeness of my Angels: The Nurses’

Participants consistently highlighted the essential emotional and physical support provided by nurses.

KP11 noted:

Every time you heard a noise, they were already there, ready to intervene and calm you… they were always there, day and night.

The perception of nurses as emotionally available and anticipatory was central to the ICU experience.

OL 15 said:

They were always ready, almost anticipating my requests… I called them my angels, they knew what you needed before you even said it.

Such descriptions affirm the dual role of nurses as both clinical caregivers and emotional anchors.

### 5.5 THEME 5: ‘The Other Side: Exclusion From Care’

Despite the strong support from nurses, many patients felt excluded from clinical conversations and decisions.

QJ 17 said:

The only thing that made me really angry was when they talked about me without even looking at me… I didn't understand all those terms, but if explained differently, I could have understood from a patient's point of view… I'm there, you tell me when to drink, how to eat, but you can't ask me if I feel okay in this position?

This quote exemplifies the patients' desire to be acknowledged as active agents in their own care.

SH 19 explained:

Sometimes you became invisible to them… they moved you, touched you, talked among themselves… as if you were in another room… hey, I'm here, ask me, talk to me.

These reflections reveal the psychological toll of passive roles and highlight the need for more inclusive care models.

## 6 Discussion

This study provides valuable insights into the lived experiences of patients in the ICU after cardiac surgery, highlighting themes that resonate with the existing literature while uncovering new areas for further investigation.

Consistent with previous research, our study found that fear and anxiety were predominant emotions experienced by patients undergoing cardiac surgery. This aligns with the findings of Wong and Arthur (2000) in Hong Kong [23], who documented high levels of anxiety and pain among ICU patients. Although their study focussed on anxiety and pain, our findings suggest that fear—specifically the fear of not waking up from surgery—can intensify both anxiety and the perception of pain. This anticipatory fear was a recurring theme among our participants and is closely related to the severe psychological distress described by Gélinas (Canada) [42], which often accompanies severe pain during the ICU stay.

Additional studies corroborate these findings. For instance, Poole et al. (UK) [60] found that greater preoperative concern about illness was significantly related to longer ICU stays in patients undergoing CABG surgery. This underscores the importance of addressing psychological distress before surgery to improve post‐operative outcomes. Although addressing psychological distress is particularly challenging in emergent cases, strategies such as rapid psychological assessment and immediate support interventions can help mitigate these effects even in urgent settings [61, 62, 63, 64]. The confusion and disoriented memories reported by our participants are supported by findings from Chahraoui et al. (France) [24], who observed that many patients had fragmented or unclear memories of their ICU stay. This confusion is often compounded by the use of sedatives and mechanical ventilation, which can impede effective communication and exacerbate feelings of helplessness, as also noted by Laitinen (Finland) [40]. Research by Cserép et al. (Hungary) [65] further supports these findings, showing that lower self‐rated health and happiness were associated with longer ICU stays and more post‐operative complications. These psychosocial variables are critical in influencing recovery trajectories.

Our participants' perception that time stood still in the ICU mirrors findings from Albanesi et al. (Italy) [11], who noted that ICU patients often experience a distorted sense of time. This phenomenon, described as ‘endless time’, contributes to the psychological burden of the ICU experience. Several factors contribute to this perception, including sleep deprivation, constant stimuli from monitors and alarms, and lack of natural light. These conditions disrupt patients' circadian rhythms and can lead to stress and memory distortions [11, 41, 66]. Therefore, modifying environmental factors should be a priority in ICU settings to reduce these negative psychological effects.

The pivotal role of nurses in providing emotional support and a sense of security emerged strongly from our findings. Strahan and Brown (UK) [25] similarly highlighted how nurse–patient interactions alleviate feelings of vulnerability. Participants often referred to nurses as ‘angels’, underscoring their appreciation. However, recent literature from Australia [67] critiques this ‘angel’ narrative, arguing that it may create unrealistic expectations and obscure the technical and emotional labour of nursing. Therefore, while recognising the human connection, it is essential to value nursing as a skilled and professional discipline [68, 69].

Demircelik et al. (Turkey) [70] reported that psychological nursing interventions significantly reduced anxiety and depression in coronary ICU patients. Furthermore, guidelines from the UK's National Institute for Health and Care Excellence [71] recommend psychological screening for anxiety, depression and PTSD to mitigate long‐term distress and improve recovery outcomes. These findings support integrating structured psychological assessments into ICU protocols as both a clinical and cost‐effective strategy.

The psychological well‐being of families also plays a vital role in patient recovery. Farzadmehr et al. (Iran) [72] showed that family support and counselling significantly reduce stress and enhance satisfaction with care. Thus, including families in support interventions not only benefits the family unit but also indirectly enhances patient recovery.

Pain management remains a critical issue, as highlighted by our participants who reported discomfort from surgical procedures and devices such as chest tubes and endotracheal tubes. This is consistent with studies by Milgrom et al. (USA) [73] and Puntillo (USA) [74], who documented high pain levels in ICU patients. Mahesh et al. (UK) [75] further found that prolonged ICU stays, often associated with unmanaged pain, predict poorer long‐term survival.

These findings highlight the need for personalised educational and support programmes in ICUs. As Alanazi (Saudi Arabia) [61], Kalogianni et al. (Greece) [62] and Guo et al. (China) [63] have demonstrated, preoperative education can significantly reduce anxiety and post‐operative complications. Such interventions, coupled with post‐ICU psychological support, form a comprehensive model of care. It seems clear that enhancing the role of nurses in providing emotional support and involving patients in care decisions can foster a more positive ICU experience [76, 77].

In addition to structured support during ICU stays, psychological interventions are necessary even after discharge. Auer et al. (Germany) [64] demonstrated that optimising preoperative expectations reduced hospital stay duration and improved recovery. Implementing similar models across healthcare systems could yield both clinical and economic benefits.

## 7 Limitations

Although this study offers valuable insights, it does have some limitations. The sample size was restricted to a specific geographic area, which may limit the diversity of perspectives. Future research should consider including a broader patient population and exploring the long‐term psychological effects of ICU stays after cardiac surgery.

## 8 Implications for Practice and Further Research

This study underscores the critical need for improved psychological support and patient‐centred communication strategies in ICUs, particularly for individuals recovering from major cardiac surgery. Implementing preoperative education programmes—delivered primarily by nurses with expertise in cardiac and critical care, and supported by psychologists and surgical team members—to set realistic expectations, alongside offering psychological interventions, could reduce anxiety and improve patient outcomes.

The unique experience of cardiac surgery patients—as distinct from general surgical populations—demands tailored support interventions. Special attention should also be directed towards patients with prolonged ICU stays, who are at greater risk of reduced quality of life, as highlighted by Diab et al. (UK) [78] and Barrie et al. (Canada) [79].

Additionally, future research should investigate the presence, predictors and trajectory of post‐intensive care syndrome in both patients and caregivers. Systematic reviews by Gravante et al. (Italy) [80] and Hwang (USA) [81] call for integrated interventions addressing the physical, psychological and cognitive sequelae of ICU stays.

Longitudinal studies exploring evolving patient needs and outcomes over time are also warranted. Moreover, as emphasised by Iaccarino et al. (Italy) [12], gender‐based differences in ICU experiences and outcomes should not be overlooked. Addressing these differences through gender‐sensitive care models may lead to more equitable and effective support pathways.

Together, these insights highlight the urgency of developing integrative, individualised and continuous psychological care approaches for ICU patients before, during and after hospitalisation.

## 9 Conclusions

In conclusion, this study underscores the complex and multifaceted experiences of ICU patients following cardiac surgery. The identified themes ‘Closing the eyes and not opening them’, ‘Confusion upon awakening’, ‘Time stood still’, ‘The closeness of my angels: the nurses’ and ‘The other side: exclusion from care’ provide a comprehensive understanding of lived experience by these patients. Addressing these issues through targeted interventions and enhanced communication can significantly improve the ICU experience and overall recovery of cardiac surgery patients.

## Author Contributions

Conceptualisation: S.S., C.M. and P.D. Methodology: C.M., V.G., V.B. and S.S. Software: R.N. and P.D. Validation: C.M., V.G. and V.B. Formal analysis: C.M., V.B., A.G. and S.S. Investigation: C.M. and V.B. Data curation: T.R. and P.D. Writing – original draft preparation: C.M., R.N., V.B. and S.S. Writing – review and editing: P.D., S.S. and T.R. Visualisation: P.D., A.G. and S.S. Supervision: P.D., S.S. and R.N. All authors have read and agreed to the published version of the manuscript. All authors approved the final manuscript as submitted and agree to be accountable for all aspects of the work.

## Ethics Statement

The study was approved by Ethics Committee of the Calabria Region, Central Area, with protocol number 276/2022 on 15 September 2022 and was performed in accordance with the Helsinki Declaration (Fortaleza revision, 2013), the Good Clinical Practice Standards (CPMP/ICH/135/95) and with the pertinent European and Italian regulations about privacy.

## Consent

Written informed consent was obtained from each subject.

## Conflicts of Interest

The authors declare no conflicts of interest.

## Data Availability

The data that support the findings of this study are available from the corresponding author upon reasonable request.

## References

[1] A. Watanabe and S. M. Wiseman , “A New Era in Surgical Research: The Evolving Role of Artificial Intelligence,” American Journal of Surgery 226 (2023): 923–925, 10.1016/j.amjsurg.2023.06.040. 37419728

[2] H. Nelson , “Surgical Innovation,” British Journal of Surgery 100 (2013): 28–30, 10.1002/bjs.9093_1. 23436716

[3] C. Weissman and N. Klein , “Who Receives Postoperative Intensive and Intermediate Care?,” Journal of Clinical Anesthesia 20 (2008): 263–270, 10.1016/j.jclinane.2007.11.005. 18617123

[4] H. Kehlet and J. B. Dahl , “Anaesthesia, Surgery, and Challenges in Postoperative Recovery,” Lancet 362 (2003): 1921–1928, 10.1016/S0140-6736(03)14966-5. 14667752

[5] J. C. Marshall , L. Bosco , N. K. Adhikari , et al., “What Is an Intensive Care Unit? A Report of the Task Force of the World Federation of Societies of Intensive and Critical Care Medicine,” Journal of Critical Care 37 (2017): 270–276, 10.1016/j.jcrc.2016.07.015. 27612678

[6] G. E. Weissman , M. P. Kerlin , Y. Yuan , et al., “Potentially Preventable Intensive Care Unit Admissions in the United States, 2006–2015,” Annals of the American Thoracic Society 17 (2020): 81–88, 10.1513/AnnalsATS.201905-366OC. 31581801 PMC6944341

[7] T.‐K. Oh , H.‐G. Kim , and I.‐A. Song , “Epidemiologic Study of Intensive Care Unit Admission in South Korea: A Nationwide Population‐Based Cohort Study From 2010 to 2019,” International Journal of Environmental Research and Public Health 20, no. 1 (2022): 81, 10.3390/ijerph20010081. 36612396 PMC9819529

[8] L. Ling , C. M. Ho , P. Y. Ng , et al., “Characteristics and Outcomes of Patients Admitted to Adult Intensive Care Units in Hong Kong: A Population Retrospective Cohort Study From 2008 to 2018,” Journal of Intensive Care 9 (2021): 2, 10.1186/s40560-020-00513-9. 33407925 PMC7788755

[9] “Census and Statistics Department,” accessed September 7, 2024, https://www.censtatd.gov.hk/en/.

[10] M. Claeys , F. Roubille , G. Casella , et al., “Organization of Intensive Cardiac Care Units in Europe: Results of a Multinational Survey,” European Heart Journal Acute Cardiovascular Care 9 (2020): 993–1001, 10.1177/2048872619883997. 31976740

[11] B. Albanesi , T. Nania , S. Barello , et al., “Lived Experience of Patients in icu After Cardiac Surgery: A Phenomenological Study,” Nursing in Critical Care 27 (2022): 204–213, 10.1111/nicc.12562. 33063374

[12] G. Iaccarino , G. Grassi , C. Borghi , et al., “Gender Differences in Predictors of Intensive Care Units Admission Among COVID‐19 Patients: The Results of the SARS‐RAS Study of the Italian Society of Hypertension,” PLoS One 15 (2020): e0237297, 10.1371/journal.pone.0237297. 33022004 PMC7537902

[13] J. B. Knight , E. E. Lebovitz , T. A. Gelzinis , and I. A. Hilmi , “Preoperative Risk Factors for Unexpected Postoperative Intensive Care Unit Admission: A Retrospective Case Analysis,” Anaesthesia Critical Care & Pain Medicine 37 (2018): 571–575, 10.1016/j.accpm.2018.02.002. 29455034

[14] A. Vijenthira , N. Chiu , D. Jacobson , et al., “Predictors of Intensive Care Unit Admission in Patients With Hematologic Malignancy,” Scientific Reports 10 (2020): 21145, 10.1038/s41598-020-78114-7. 33273653 PMC7713054

[15] H. Adamson , M. Murgo , M. Boyle , S. Kerr , M. Crawford , and D. Elliott , “Memories of Intensive Care and Experiences of Survivors of a Critical Illness: An Interview Study,” Intensive & Critical Care Nursing 20 (2004): 257–263, 10.1016/j.iccn.2004.06.005. 15450614

[16] J. A. Alasad , N. Abu Tabar , and M. M. Ahmad , “Patients' Experience of Being in Intensive Care Units,” Journal of Critical Care 30 (2015): 859.e7–859.e11, 10.1016/j.jcrc.2015.03.021. 25865939

[17] M. B. Happ , K. Garrett , D. D. Thomas , et al., “Nurse‐Patient Communication Interactions in the Intensive Care Unit,” American Journal of Critical Care 20 (2011): e28–e40, 10.4037/ajcc2011433. 21362711 PMC3222584

[18] J. Sareen , K. Olafson , M. S. Kredentser , et al., “The 5‐Year Incidence of Mental Disorders in a Population‐Based ICU Survivor Cohort,” Critical Care Medicine 48 (2020): e675–e683, 10.1097/CCM.0000000000004413. 32697508

[19] K. Olafson , R. A. Marrie , J. M. Bolton , et al., “The 5‐Year Pre‐ and Post‐Hospitalization Treated Prevalence of Mental Disorders and Psychotropic Medication Use in Critically Ill Patients: A Canadian Population‐Based Study,” Intensive Care Medicine 47 (2021): 1450–1461, 10.1007/s00134-021-06513-z. 34495357

[20] G. S. Bhogale , R. B. Nayak , M. Dsouza , S. S. Chate , and M. B. Banahatti , “A Cross‐Sectional Descriptive Study of Prevalence and Nature of Psychiatric Referrals From Intensive Care Units in a Multispecialty Hospital,” Indian Journal of Psychological Medicine 33 (2011): 167–171, 10.4103/0253-7176.92063. 22345844 PMC3271494

[21] G. Zisopoulos , P. Roussi , and E. Mouloudi , “Psychological Morbidity a Year After Treatment in Intensive Care Unit,” Health Psychology Research 8 (2020): 8852, 10.4081/hpr.2020.8852. 33553785 PMC7859955

[22] W. W. Geense , M. Zegers , M. A. A. Peters , et al., “New Physical, Mental, and Cognitive Problems 1 Year After ICU Admission: A Prospective Multicenter Study,” American Journal of Respiratory and Critical Care Medicine 203 (2021): 1512–1521, 10.1164/rccm.202009-3381OC. 33526001

[23] F. Y. K. Wong and D. G. Arthur , “Hong Kong Patients' Experiences of Intensive Care After Surgery: Nurses' and Patients' Views,” Intensive & Critical Care Nursing 16 (2000): 290–303, 10.1054/iccn.2000.1515. 11000603

[24] K. Chahraoui , A. Laurent , A. Bioy , and J.‐P. Quenot , “Psychological Experience of Patients 3 Months After a Stay in the Intensive Care Unit: A Descriptive and Qualitative Study,” Journal of Critical Care 30 (2015): 599–605, 10.1016/j.jcrc.2015.02.016. 25776895

[25] E. Strahan and R. J. Brown , “A Qualitative Study of the Experiences of Patients Following Transfer From Intensive Care,” Intensive & Critical Care Nursing 21 (2005): 160–171, 10.1016/j.iccn.2004.10.005. 15907668

[26] S. Bench and T. Day , “The User Experience of Critical Care Discharge: A Meta‐Synthesis of Qualitative Research,” International Journal of Nursing Studies 47 (2010): 487–499, 10.1016/j.ijnurstu.2009.11.013. 20004396

[27] A. Gullberg , E. Joelsson‐Alm , and A. Schandl , “Patients' Experiences of Preparing for Transfer From the Intensive Care Unit to a Hospital Ward: A Multicentre Qualitative Study,” Nursing in Critical Care 28 (2023): 863–869, 10.1111/nicc.12855. 36325990

[28] M. Danielis , A. Povoli , E. Mattiussi , and A. Palese , “Understanding Patients' Experiences of Being Mechanically Ventilated in the Intensive Care Unit: Findings From a Meta‐Synthesis and Meta‐Summary,” Journal of Clinical Nursing 29 (2020): 2107–2124, 10.1111/jocn.15259. 32243007

[29] S. Simeone , M. Perrone , G. Dell'Angelo , et al., “Ventilazione Meccanica ed Estubazione Precoce: Studio Qualitativo Sul Vissuto Dei Degenti di Una Terapia Intensiva Cardiochirurgia,” Assistenza Infermieristica e Ricerca 34, no. 4 (2016): 188–193, 10.1702/2110.22862. 26779875

[30] J. L. Y. Tsang , K. Ross , F. Miller , et al., “Qualitative Descriptive Study to Explore Nurses' Perceptions and Experience on Pain, Agitation and Delirium Management in a Community Intensive Care Unit,” BMJ Open 9 (2019): e024328, 10.1136/bmjopen-2018-024328. PMC6500293 30948568

[31] S. Mercadante , C. Gregoretti , and A. Cortegiani , “Palliative Care in Intensive Care Units: Why, Where, What, Who, When, How,” BMC Anesthesiology 18 (2018): 106, 10.1186/s12871-018-0574-9. 30111299 PMC6094470

[32] N. Dağcan , D. Özden , and G. Gürol Arslan , “Pain Perception of Patients in Intensive Care Unit After Cardiac Surgery: A Qualitative Study Using Roy's Adaptation Model,” Nursing in Critical Care 29 (2024): 512–520, 10.1111/nicc.12958. 37527978

[33] N. Dağcan Şahin and G. Gürol Arslan , “Perspectives of Patients, Families and Nurses on Pain After Cardiac Surgery: A Qualitative Study,” Nursing in Critical Care 29 (2024): 501–511, 10.1111/nicc.13000. 37970732

[34] D. Vervoort , B. Meuris , B. Meyns , and P. Verbrugghe , “Global Cardiac Surgery: Access to Cardiac Surgical Care Around the World,” Journal of Thoracic and Cardiovascular Surgery 159 (2020): 987–996.e6, 10.1016/j.jtcvs.2019.04.039. 31128897

[35] H. Ohbe , H. Matsui , R. Kumazawa , and H. Yasunaga , “Postoperative ICU Admission Following Major Elective Surgery: A Nationwide Inpatient Database Study,” European Journal of Anaesthesiology 39 (2022): 436–444, 10.1097/EJA.0000000000001612. 34636358

[36] V. Trivedi , H. Bleeker , N. Kantor , S. Visintini , D. I. McIsaac , and B. McDonald , “Survival, Quality of Life, and Functional Status Following Prolonged ICU Stay in Cardiac Surgical Patients: A Systematic Review,” Critical Care Medicine 47 (2019): e52–e63, 10.1097/CCM.0000000000003504. 30398978

[37] O. P. Heimrath , K. J. Buth , and J.‐F. Légaré , “Long‐Term Outcomes in Patients Requiring Stay of More Than 48 Hours in the Intensive Care Unit Following Coronary Bypass Surgery,” Journal of Critical Care 22 (2007): 153–158, 10.1016/j.jcrc.2006.09.009. 17548027

[38] S. V. Desai , T. J. Law , and D. M. Needham , “Long‐Term Complications of Critical Care,” Critical Care Medicine 39 (2011): 371–379, 10.1097/CCM.0b013e3181fd66e5. 20959786

[39] S. Valdix and K. Puntillo , “Pain, Pain Relief and Accuracy of Their Recall After Cardiac Surgery,” Progress in Cardiovascular Nursing 10 (1995): 3–11. 7479660

[40] H. Laitinen , “Patients' Experience of Confusion in the Intensive Care Unit Following Cardiac Surgery,” Intensive & Critical Care Nursing 12 (1996): 79–83, 10.1016/S0964-3397(96)80994-3. 8845628

[41] J. Grimm , “Sleep Deprivation in the Intensive Care Patient,” Critical Care Nurse 40 (2020): e16–e24, 10.4037/ccn2020939. 32236431

[42] C. Gélinas , “Management of Pain in Cardiac Surgery ICU Patients: Have we Improved Over Time?,” Intensive & Critical Care Nursing 23 (2007): 298–303, 10.1016/j.iccn.2007.03.002. 17448662

[43] A. Karasz , K. Dempsey , and R. Fallek , “Cultural Differences in the Experience of Everyday Symptoms: A Comparative Study of South Asian and European American Women,” Culture, Medicine and Psychiatry 31 (2007): 473–497, 10.1007/s11013-007-9066-y. 17985219

[44] K. H. Gattario , A. Frisén , T. L. Teall , and N. Piran , “Embodiment: Cultural and Gender Differences and Associations With Life Satisfaction,” Body Image 35 (2020): 1–10, 10.1016/j.bodyim.2020.07.005. 32877841

[45] M. Cohen , D. Kahn , and R. Steeves , Hermeneutic Phenomenological Research: A Practical Guide for Nurse Researchers (SAGE Publications, Inc., 2000).

[46] D. Tuohy , A. Cooney , M. Dowling , K. Murphy , and J. Sixsmith , “An Overview of Interpretive Phenomenology as a Research Methodology,” Nurse Researcher 20 (2013): 17–20, 10.7748/nr2013.07.20.6.17.e315. 23909107

[47] A. Flood , “Understanding Phenomenology: Anne Flood Looks at the Theory and Methods Involved in Phenomenological Research,” Nurse Researcher 17 (2010): 7–15, 10.7748/nr2010.01.17.2.7.c7457. 20222274

[48] C. Moretti , S. E. Ceccaroni , R. Confortini , et al., “Taking Care. Nursing Towards Covid‐19 Patients During the Pandemic Emergency in Italy: A Qualitative Study,” Acta Biomedica Atenei Parmensis 92, no. S2 (2021): e2021025, 10.23750/abm.v92iS2.11944. PMC8383221 34328132

[49] E. Vellone , N. Sinapi , and D. Rastelli , “Phenomenology and Phenomenological Method: Their Usefulness for Nursing Knowledge and Practice,” Professioni Infermieristiche 53 (2000): 237–242. 12424957

[50] A. Tong , P. Sainsbury , and J. Craig , “Consolidated Criteria for Reporting Qualitative Research (COREQ): A 32‐Item Checklist for Interviews and Focus Groups,” International Journal for Quality in Health Care 19 (2007): 349–357, 10.1093/intqhc/mzm042. 17872937

[51] D. Polit and C. Beck , “Essentials of Nursing Research,” Ethics 23 (2012): 145–160.

[52] E. Vellone , G. Piras , G. Venturini , R. Alvaro , and M. Z. Cohen , “The Experience of Quality of Life for Caregivers of People With Alzheimer's Disease Living in Sardinia, Italy,” Journal of Transcultural Nursing 23 (2012): 46–55, 10.1177/1043659611414199. 21807960

[53] J. Phillippi and J. Lauderdale , “A Guide to Field Notes for Qualitative Research: Context and Conversation,” Qualitative Health Research 28 (2018): 381–388, 10.1177/1049732317697102. 29298584

[54] D. F. Polit and C. T. Beck , Essentials of Nursing Research: Appraising Evidence for Nursing Practice (Lippincott Williams & Wilkins, 2010).

[55] A. Strauss and J. Corbin , Basics of Qualitative Research: Techniques and Procedures for Developing Grounded Theory, 2nd ed. (Sage Publications, Inc, 1998).

[56] Y. S. Lincoln and E. G. Guba , “But Is It Rigorous? Trustworthiness and Authenticity in Naturalistic Evaluation,” New Directions for Program Evaluation 1986 (1986): 73–84, 10.1002/ev.1427.

[57] A. K. Shenton , “Strategies for Ensuring Trustworthiness in Qualitative Research Projects,” Education for Information 22 (2004): 63–75, 10.3233/EFI-2004-22201.

[58] J. Gunawan , “Ensuring Trustworthiness in Qualitative Research,” Belitung Nursing Journal 1 (2015): 10–11, 10.33546/bnj.4.

[59] World Health Organization , “Process of Translation and Adaptation of Instruments,” 2009, http://www.who.int/substance_abuse/research_tools/translation/en/.

[60] L. Poole , T. Kidd , E. Leigh , A. Ronaldson , M. Jahangiri , and A. Steptoe , “Psychological Distress and Intensive Care Unit Stay After Cardiac Surgery: The Role of Illness Concern,” Health Psychology 34 (2015): 283–287, 10.1037/hea0000183. 25528184

[61] A. A. Alanazi , “Reducing Anxiety in Preoperative Patients: A Systematic Review,” British Journal of Nursing 23 (2014): 387–393, 10.12968/bjon.2014.23.7.387. 24732993

[62] A. Kalogianni , P. Almpani , L. Vastardis , G. Baltopoulos , C. Charitos , and H. Brokalaki , “Can Nurse‐Led Preoperative Education Reduce Anxiety and Postoperative Complications of Patients Undergoing Cardiac Surgery?,” European Journal of Cardiovascular Nursing 15 (2016): 447–458, 10.1177/1474515115602678. 26304701

[63] P. Guo , L. East , and A. Arthur , “A Preoperative Education Intervention to Reduce Anxiety and Improve Recovery Among Chinese Cardiac Patients: A Randomized Controlled Trial,” International Journal of Nursing Studies 49 (2012): 129–137, 10.1016/j.ijnurstu.2011.08.008. 21943828

[64] C. J. Auer , J. A. C. Laferton , M. C. Shedden‐Mora , S. Salzmann , R. Moosdorf , and W. Rief , “Optimizing Preoperative Expectations Leads to a Shorter Length of Hospital Stay in CABG Patients: Further Results of the Randomized Controlled PSY‐HEART Trial,” Journal of Psychosomatic Research 97 (2017): 82–89, 10.1016/j.jpsychores.2017.04.008. 28606503

[65] Z. Cserép , E. Losoncz , R. Tóth , et al., “Self‐Rated Health Is Associated With the Length of Stay at the Intensive Care Unit and Hospital Following Cardiac Surgery,” BMC Cardiovascular Disorders 14 (2014): 171, 10.1186/1471-2261-14-171. 25432074 PMC4258288

[66] T. Fukuda , Y. Kinoshita , T. Shirahama , S. Miyazaki , N. Watanabe , and T. Misawa , “Distorted Memories and Related Factors in ICU Patients,” Clinical Nursing Research 31 (2022): 39–45, 10.1177/1054773820980162. 33289396

[67] J. Stokes‐Parish , D. Barrett , R. Elliott , D. Massey , K. Rolls , and N. Credland , “Fallen Angels and Forgotten Heroes: A Descriptive Qualitative Study Exploring the Impact of the Angel and Hero Narrative on Critical Care Nurses,” Australian Critical Care 36 (2023): 3–9, 10.1016/j.aucc.2022.11.008. 36470775 PMC9716433

[68] K. Blomberg , P. Griffiths , Y. Wengström , C. May , and J. Bridges , “Interventions for Compassionate Nursing Care: A Systematic Review,” International Journal of Nursing Studies 62 (2016): 137–155, 10.1016/j.ijnurstu.2016.07.009. 27494429

[69] P. S. Groves , J. L. Bunch , and F. Kuehnle , “Increasing a Patient's Sense of Security in the Hospital: A Theory of Trust and Nursing Action,” Nursing Inquiry 30 (2023): e12569, 10.1111/nin.12569. 37282711

[70] M. B. Demircelik , M. Cakmak , Y. Nazli , et al., “Effects of Multimedia Nursing Education on Disease‐Related Depression and Anxiety in Patients Staying in a Coronary Intensive Care Unit,” Applied Nursing Research 29 (2016): 5–8, 10.1016/j.apnr.2015.03.014. 26856480

[71] “Overview|Rehabilitation After Critical Illness in Adults|Quality Standards|NICE,” accessed September 10, 2024, https://www.nice.org.uk/guidance/qs158.

[72] M. Farzadmehr , M. Fallahi‐khoshknab , M. Hosseini , H. R. Khankeh , and Z. Noorabadi , “The Effect of Nursing Consultation on Satisfaction of Patients' Families at the Cardiac Surgery Intensive Care Units,” Iranian Journal of Rehabilitation Research in Nursing 2, no. 1 (2015): 35–44.

[73] L. B. Milgrom , J. A. Brooks , R. Qi , K. Bunnell , S. Wuestefeld , and D. Beckman , “Pain Levels Experienced With Activities After Cardiac Surgery,” American Journal of Critical Care 13 (2004): 116–125, 10.4037/ajcc2004.13.2.116. 15043239

[74] K. Puntillo , “Dimensions of Procedural Pain and Its Analgesic Management in Critically Ill Surgical Patients,” American Journal of Critical Care 3 (1994): 116–122. 7513228

[75] B. Mahesh , C. K. Choong , K. Goldsmith , C. Gerrard , S. A. M. Nashef , and A. Vuylsteke , “Prolonged Stay in Intensive Care Unit Is a Powerful Predictor of Adverse Outcomes After Cardiac Operations,” Annals of Thoracic Surgery 94 (2012): 109–116, 10.1016/j.athoracsur.2012.02.010. 22579949

[76] R. Naef , S. Von Felten , H. Petry , J. Ernst , and P. Massarotto , “Impact of a Nurse‐Led Family Support Intervention on Family Members' Satisfaction With Intensive Care and Psychological Wellbeing: A Mixed‐Methods Evaluation,” Australian Critical Care 34 (2021): 594–603, 10.1016/j.aucc.2020.10.014. 33637427

[77] G. Gardner , D. Elliott , J. Gill , M. Griffin , and M. Crawford , “Patient Experiences Following Cardiothoracic Surgery: An Interview Study,” European Journal of Cardiovascular Nursing 4 (2005): 242–250, 10.1016/j.ejcnurse.2005.04.006. 15923146

[78] M. S. Diab , R. Bilkhu , G. Soppa , et al., “The Influence of Prolonged Intensive Care Stay on Quality of Life, Recovery, and Clinical Outcomes Following Cardiac Surgery: A Prospective Cohort Study,” Journal of Thoracic and Cardiovascular Surgery 156 (2018): 1906–1915.e3, 10.1016/j.jtcvs.2018.05.076. 30336918

[79] K. Barrie , A. Cornick , S. Debreuil , et al., “Patients With a Prolonged Intensive Care Unit Length of Stay Have Decreased Health‐Related Quality of Life After Cardiac Surgery,” Seminars in Thoracic and Cardiovascular Surgery 31 (2019): 21–31, 10.1053/j.semtcvs.2018.07.005. 30012367

[80] F. Gravante , F. Trotta , S. Latina , et al., “Quality of Life in icu Survivors and Their Relatives With Post‐Intensive Care Syndrome: A Systematic Review,” Nursing in Critical Care 29 (2024): 807–823, 10.1111/nicc.13077. 38622971

[81] D. Y. Hwang , “Mitigating Postintensive Care Syndrome Among Patients and Caregivers via a Dyadic Intervention,” JAMA Network Open 3 (2020): e2021014, 10.1001/jamanetworkopen.2020.21014. 33052398

